# Evaluation of early-generation tropical maize testcrosses for grain-yield potential and weevil (*Sitophilus zeamais* Motschulsky) resistance

**DOI:** 10.1016/j.cropro.2020.105384

**Published:** 2021-01

**Authors:** Julius P. Sserumaga, Dan Makumbi, Sylvester O. Oikeh, Michael Otim, Lewis Machida, Bruce Y. Anani, Egas Nhamucho, Yoseph Beyene, Stephen Mugo

**Affiliations:** a National Agricultural Research Organization, National Livestock Resources Research Institute, P.O. Box 5407, Kampala, Uganda; b International Maize and Wheat Improvement Center (CIMMYT), P.O. Box 1041-00621, Nairobi, Kenya; c AATF, P.O. Box 30709-00100, Nairobi, Kenya; d National Agricultural Research Organization, National Crops Resources Research Institute, Namulonge, P.O. Box 7084, Kampala, Uganda; e Mozambique Agricultural Research Institute (IIAM), P.O. Box 3658, FPLM Av. N., 2698, Maputo, Mozambique

**Keywords:** Stress, Maize weevil, Post-harvest, Heritability

## Abstract

Smallholder maize farmers in Africa experience pre- and post-harvest production stresses either individually or in combination at different stages of the crop cycle. The maize weevil is among the major post-harvest storage pests. A strategy to address this problem is to develop and promote high yielding maize germplasm with resistance to multiple stresses. A study was conducted to: 1) assess yield and agronomic performance of testcross hybrids developed from early generation lines; and 2) assess the response of the testcross hybrids to infestation with *Sitophilus zeamais.* Fifty-eight drought-tolerant testcross hybrids were evaluated for agronomic performance and weevil resistance at four environments in Uganda in 2016. Hybrid G39 (L2/T2) had the best grain yield performance; it significantly out-performed the best check by 11.4% in all environments. Hybrid grain from field trials was subjected to *Sitophilus zeamais* infestation in a choice and no choice test under laboratory conditions. Hybrids G56 (L49/T2) and G58 (L51/T2) had the least weevil damage and were rated as resistant to *Sitophilus zeamais*. The numbers of damaged kernels, number of exit holes and ear aspect were positively correlated with the grain weight loss. The results suggest possibilities for simultaneous selection for high grain yield and storage insect pest resistance among drought-tolerant genotypes. Use of high-yielding and resistant maize hybrids to storage insect pest should be promoted for increased maize production and managing post-harvest losses due to the maize weevil in smallholder farming communities in Africa.

## Highlights

• Currently there is no effective a single bullet strategy to address pre- and post-harvest production stresses due to insect damage in Africa.
• Fifty eight drought-tolerant experimental testcrosses hybrids were evaluated for agronomic performance and weevil resistance.
• Hybrids G56 (L49/T2) and G58 (L51/T2) had the least weevil damage and were rated as resistant to *S. zeamais.*

## 1 Introduction

In Sub-Saharan Africa (SSA), maize (*Zea mays* L.) is the most important staple crop among the five biggest crops that contribute more than 45% of total crop production value (OECD/FAO, 2018). In Uganda, average per capita consumption was estimated to be 415 kcal/capita/day (FAOSTAT, 2016). Although maize is considered to be an important crop in eastern African, there is still a deficit in production of the staple due to low soil fertility, frequent droughts, and insect pest damage. Smallholder maize farmers in eastern Africa experience pre- and post-harvest production stresses either individually or in combination at different stages of the crop cycle. The maize production deficit is aggravated by overwhelming post-harvest losses. Most important economic quantifiable post-harvest losses occurs in the field (15%), during storage (15%–25%), and during processing (13%–20%) (Abass et al., 2014). Among other storage pests, grain weevils (*Sitophilus zeamais* and *S. granarius*) and larger grain borers (*Prostephanus truncatus*) are responsible for the major losses (Abass et al., 2014).

Losses of up to 15%–90% among smallholder farmers (Tefera et al., 2011) are attributed to the maize weevil hence ranking it among the most destructive storage pests of maize grain in the tropical and subtropical regions of the world. *S. zeamais* infestation in the storage leads to reduction of quantities of grains and lower nutritional and market values of the grains, and thus increases poverty (Keba and Sori, 2013). Additionally, *S. zeamais* infestation affects percentage germination which results in low production since seed and grains are stored together (Pingali, 2001).

Several control options including chemical and cultural can be used to reduce post-harvest losses due to maize weevil damage in stored grain. The major control of weevils has been the use of chemicals; however, reports indicated the rise of insecticide-resistant *S. zeamais* populations (Asawalam and Hassanali, 2006; Guedes et al., 1995; Kljajić and Perić, 2006; Oliveira et al., 2007; Ribeiro et al., 2003). Pesticides are expensive and have residual effects and there is, therefore, a call for cheaper and environmentally safer options for maize weevil control. There is a need to breed hybrids with good levels of resistance to safeguard farmers from loss. Resistant varieties can be incorporated in any insect management program because they are friendly to the environment, effective and safe (Keba and Sori, 2013). Flint maize offer promise to weevil control since their outer layers makes it less susceptible to insect damage and mould colonization in the fields and during storage (Suleiman et al., 2015).

The need for weevil-resistant maize varieties has implications for germplasm development efforts in eastern Africa as breeders need to concurrently select for abiotic stress tolerance and weevil resistance during line development. In the last two decades, the National Agricultural Research Organization (NARO) in collaboration with the International Maize and Wheat Improvement Center (CIMMYT) have developed elite maize genotypes with tolerance to multiple stresses (drought, maize streak virus, turcicum leaf blight, gray leaf spot, and *Striga*) using new elite inbred lines (Beyene et al., 2013; Makumbi et al., 2015; Sserumaga et al., 2018, 2016). Previous evaluation of new stress tolerant germplasm (Beyene et al., 2013; Sserumaga et al., 2018, 2016) did not consider assessment for weevil resistance. There is need to combine selection for multiple traits during the early stages of inbred line development. Breeding efficiency can be improved through early generation selection thus reducing the number of genotypes in subsequent trials, cost of trials, and in turn increasing genetic gain per unit cost (Fischer and Rebetzke, 2018). To develop superior hybrids that can improve productivity, new genotypes should be evaluated for performance on all desirable traits during early stages to identify genotypes that combine high yield and maize weevil resistance. The aims of this study were to: 1) assess yield and agronomic performance of testcross hybrids developed from early-generation lines; and 2) assess the response of the testcross hybrids to infestation with *Sitophilus zeamais.*

## 2 Materials and methods

### 2.1 Trial materials

Forty-three (43) F_2:3_ drought-tolerant inbred lines were selected for this study. The F1 crosses were derived from a group of elite inbred lines developed from seven tropical bi-parental crosses between drought-tolerant inbred lines from CIMMYT (Beyene et al., 2016) and three elite drought tolerant inbred lines with resistance to common African diseases from NARO (Table 1). The maize populations were advanced to F_2:3_ basing on plant type, low ear placement, yield potential, and low disease reaction to common African diseases (Vivek et al., 2010). Based on these selection criteria, 43 F_2:3_ lines were selected and testcrossed to two heterotic single-cross testers for stage one testing. Due to the difference in the nicking between the lines and testers, 58 hybrids were generated for this study.

**Table 1:** List of inbred lines used to form segregating population.

No.	Name	Pedigree	Source	Attribute
1	NML85	[KILIMA (ST94)-S5:115/[M37W/ZM607#BF37SR …]]-B-B-1-3-#-B	NARO	Drought tolerant, GLS, TLB and MSV resistant
2	NML88	[KILIMA (ST94)-S5:115/[M37W/ZM607#BF37SR …]]-B-B-1-6-#-B	NARO	Drought tolerant, GLS, TLB and MSV resistant
3	NML97	[[EV7992#/EV8449-SR]C1F2-334-1(OSU9i)-8-6(I)-X-X-1-B-B/CML206]-B-B-2-1-#-B	NARO	Drought tolerant, GLS, TLB and MSV resistant
4	CKDHL0165	(La Posta Seq C7-F96-1-2-1-1-B-B-B/CML444/CML444) DH-104-B-B-B	CIMMYT	Drought tolerant, GLS, TLB and MSV resistant
5	CKDHL0216	(La Posta Seq C7-F71-1-2-1-2-B-B-B/CML312SR = MAS [MSR/312]-117-2-2-1-2-B*4-B-B-B-B/CML312SR) DH-5-B-B-B	CIMMYT	Drought tolerant, GLS, TLB and MSV resistant
6	CKDHL0221	(La Posta Seq C7-F71-1-2-1-2-B-B-B/CML312SR = MAS [MSR/312]-117-2-2-1-2-B*4-B-B-B-B/CML312SR) DH-10-B-B-B	CIMMYT	Drought tolerant, GLS, TLB and MSV resistant
7	CKDHL0227	(La Posta Seq C7-F71-1-2-1-2-B-B-B/CML312SR = MAS [MSR/312]-117-2-2-1-2-B*4-B-B-B-B/CML312SR) DH-18-B-B-B	CIMMYT	Drought tolerant, GLS, TLB and MSV resistant
8	CKDHL0277	(La Posta Seq C7-F71-1-2-1-2-B-B-B/CML312SR = MAS [MSR/312]-117-2-2-1-2-B*4-B-B-B-B/CML312SR) DH-100-B-B-B	CIMMYT	Drought tolerant, GLS, TLB and MSV resistant
9	CKDHL0295	(La Posta Seq C7-F71-1-2-1-2-B-B-B/CML395/CML395) DH-21-B-B-B	CIMMYT	Drought tolerant, GLS, TLB and MSV resistant
10	CKDHL0333	(La Posta Seq C7-F71-1-2-1-2-B-B-B/CML395/CML395) DH-65-B-B-B	CIMMYT	Drought tolerant, GLS, TLB and MSV resistant
11	CKDHL0373	(La Posta Seq C7-F71-1-2-1-2-B-B-B/CML395/CML395) DH-107-B-B-B	CIMMYT	Drought tolerant, GLS, TLB and MSV resistant
12	CKDHL0470	(La Posta Seq C7-F71-1-2-1-2-B-B-B/CML444/CML444) DH-49-B-B-B	CIMMYT	Drought tolerant, GLS, TLB and MSV resistant

### 2.2 Trial design, crop management and data collection

Fifty-eight (58) hybrids and two popular checks were evaluated across four environments (Serere, Bulindi, Ngetta and Ikulwe) that represent some of the major maize growing agroecologies in Uganda. The soil type at Serere (1°31′N, 33°27′E, 1080 masl) is sandy clay loams and black clays, classified as Petric Plinthosol. The mean annual rainfall at Serere is 1419 mm. The soil type at Bulindi (0° 16′N, 32° 52’E’; 1144 masl) is sandy loam, classified as Acric Ferralsol. The average annual rainfall at Bulindi is 1338 mm. The soil type at Ngetta (2° 16′N, 32° 52′E; 1300 masl) is sandy loam, classified as Vertisol. The average annual rainfall at Ngetta is 1483 mm. The soil type at Ikulwe (0° 26′N, 33° 28′E; 1170 masl) is sandy loam, classified as Petric Plinthosols. The average annual rainfall at Ikulwe is 1345 mm. The rainfall distribution at all four environments is bimodal with long rains in first season (March–July) and short rains second season (September–November). The trials were planted during the long rainy season (March–August). At all sites, the entries were hand-planted in two row plots of 5 m long and spacing of 0.75 m between rows and 0.25 m between hills. The trial design was a 6 × 10 α-lattice (Patterson and Williams, 1976) with two replications at each environment. Two seeds were planted and later thinned to one plant three weeks after getting final plant population of approximately 53,333 plants/ha. All trials were kept weed free by regular hand weeding and recommended agronomic practices for every environment were followed.

Data were recorded on cob weight, days to anthesis (AD), grain texture (TEX), husk cover (HC), and ear aspect (EA) at all sites. AD was recorded by counting the number of days from planting to when 50% of plants had shed pollen. Grain texture was recorded by scoring whole ears in each plot on a scale of 1–5, where 1 = flint, round crown kernel with vitreous appearance, and 5 = dent kernel with a floury endosperm. Ear aspect (EA) was rated on a scale of 1–5 (where 1 = nice uniform ears with the preferred texture and 5 = ears with the undesirable texture). Husk cover (HC) was assessed by considering the plants with bare-tip husks; their counts per plot were expressed as proportion of the total plant population per plot.

To minimize the border effects, plants at end from either side of each row were removed at harvest. All the ears per plot were weighed individually and hand-shelled after which a representative sample of grain was collected and used to determine grain moisture employing a Dickey-John moisture meter (MINIGAC1) in the least environments. Ear weight was used to calculate for grain yield after adjusting grain moisture to 12.5% of expressed as t/ha. A grain sample of 500 gm from each plot at each environment was collected in a paper bag and transported to National Crops Resources Research Institute (NaCRRI) for assessment of reaction to weevil infestation.

### 2.3 Rearing of maize weevils for infestation

Adults of *S. zeamais* weevil maintained at National Crops Resources Research Institute (NaCRRI), Weevil Screening Laboratory in Namulonge were used in this study. In a 1 L perforated lid of glass jar, 400 g of Longe 5 (one of the susceptible open pollinated maize varieties) clean grain with moisture content 11%–12% were placed with 200 unsexed adult weevils and maintained at 27 ± 2 °C and 65%–70% relative humidity with a 12:12 (light: dark respectively) photoperiod (Adebayo and Omoloye, 2012; Guthrie et al., 1981). The weevils were left for 10 days to oviposit and later removed to allow progeny emergence and harvested when sufficient numbers were obtained.

### 2.4 Preparation of grain for weevil screening

The trials were set up at the Weevil Screening Laboratory. Grain samples from the field for each genotype at each environment were dried in paper bags to avoid direct heat on the kernels and to attain near uniform moisture content of 12%. In order to destroy adult insects and eggs that might have been present due to natural infestation in the field, grains were kept in a fumigated plastic drum containing phosphine-fumigant (Gastoxin™) (Nhamucho et al., 2017). Dust, dirt and broken grains were sieved and a sub sample (50 g) of each entry was weighed and left for 24 h in a jar at room temperature before infestation with weevils.

A no choice test was carried out on each entry. In this test, 50 mature, active and unsexed 20–25 days old adult weevils were used for infestation in a 250 ml glass jar containing 50 g of grains (Dobie, 1974; Nhamucho et al., 2017; Siwale et al., 2010). The glass jars were covered with a wire mesh lid to allow air circulation and prevent the insects from escaping. The trials were arranged following the field trial design but with two replications in a laboratory at controlled condition (27 ± 2 °C and 60 ± 10% relative humidity). The weevils were left to oviposit for 10 days and later the adults were sieved out on 1.0 mm and 4.75 mm sieves (Endecotts Ltd, UK) to separate the insect from grains plus the powder that collected in the lower pan. In the process, weevils and grains were collected on the 1.0 mm and 4.75 mm mesh respectively. The dead and live weevils were identified and counted with tweezers and a tally counter (Nhamucho et al., 2017). Tweezers was used to probe the weevils for immobility in order to establish whether they were alive or dead (Nhamucho et al., 2017; Siwale et al., 2010). Then the parent mortality was calculated from the number of dead parental stock and, using a precision electronic scale, the weight of the flour/dust produced during insect feeding and the grains was determined. The dust weight was expressed as a level of the underlying grain weight. Grains were sorted manually in order to differentiate between the undamaged and damaged ones. Those with holes and/or tunnels (damaged) caused by insects that were observed under a magnifying glass per grain; and expressed as percentage damaged kernels. Both the undamaged and insect-damaged maize grains were weighed and counted. Grain damage was computed as an extent of damaged grains over the all-out number of grains tested. The level of weight loss was evaluated utilizing tally and gauge strategy according to Boxall (1986) expressed as below:

$$Weight\;\;loss\;\;\left(\%\right)=\frac{\left(Wu*Nd\right)-\left(Wd*Nu\right)}{Wu*\left(Nd+Nu\right)}\times 100$$

where Wu = weight of undamaged grains, Wd = weight of insect-damaged grains, Nu = number of undamaged grains and Nd = number of insect-damaged grains.

### 2.5 Data analysis

#### 2.5.1 Analysis of variance for agronomic performance

Separate analysis of variance for all traits was done for every environment and combined across environments using PROC MIXED procedure of SAS (SAS, 2011). Replication and blocks within replications were considered as random effects and genotypes as fixed effects. Different sources of variation were partitioned to derive variances to check for contrasts among genotypes and the nearness of G × E association. The model used in the analysis considered environments and genotypes as random effects as described by Yamada (1962) as;

$$Y_{ijk}=\mu +{\text{G}}_{i}+{\text{A}}_{j}+{\text{GA}}_{ij}+\frac{\text{B}}{{\text{A}}_{jk}}+\varepsilon_{ijk}$$

where Y_ijk_ equates to observed value of the ith progeny of the jth environment in the kth replications, μ = general mean, G = effect of the ith genotype (i = 1, 2, .i), A: effect of the jth environment (j = 1, 2, … j), GA = effects of the interaction of the ith progeny with the jth environment, B/A_jk_ = effect of the kth block within the jth environment, and Ɛ_ijk_ = random error.

Significance test for genotype effects across environment was computed as the pooled error term using corresponding interaction with environment. Grain yield and other traits for individual environment and across analysis was computed using mixed model analysis in META-R to get best linear unbiased estimates (BLUEs) for the genotypes (Alvarado et al., 2020). For comparison of entries evaluated at several environments, the genotype means were expressed as a percentage of the standard performance of the best commercial check hybrid within the respective environments.

#### 2.5.2 Analysis of variance for weevil resistance

Using Kolmogorov-Smirnov test, data on loss in grain weight, number of damaged kernels and mortality of weevils were tested for normality and transformed before analysis. After transformation data were subjected to ANOVA using the general linear model in Genstat 15th Edition (Payne et al., 2012). Multiple means comparisons were done using Fisher's Protected LSD test. All tests were performed at α = 0.05 and Computed Pearson's correlation coefficients between traits using PROC CORR of SAS (SAS, 2011).

#### 2.5.3 Genotypic variances and heritability

Estimates of genotypic ($\sigma_{G}^{2}$), environment ($\sigma_{L}^{2}$), genotype × environment ($\sigma_{GxL}^{2}$), and error variance ($\sigma_{E}^{2}$) for field data was computed using the PROC MIXED of SAS (SAS, 2011). Broad-sense heritability was computed as per Hallauer et al. (2010) for individual trials as;

$$H=\frac{\sigma_{G}^{2}}{\left[\sigma_{G}^{2}\left\{\frac{\sigma_{E}^{2}}{r}\right\}\right]}$$

where; $\sigma_{G}^{2}$ is the genotypic variance,$\;\sigma_{E}^{2}$ is the error variance, and r the number of replications.

Across environments, broad-sense heritability for each trait was assessed using variance components as per Hallauer et al. (2010) formula below:

$$H=\frac{\sigma_{G}^{2}}{\left[\sigma_{G}^{2}+\frac{\sigma_{GxL}^{2}}{E}+\frac{\sigma_{E}^{2}}{ER}\right]}$$

where $\sigma_{G}^{2}$ = genotypic variance, $\sigma_{GxL}^{2}$ = genotype × environment and $\sigma_{E}^{2}$ = residual variance components, R is the number of replications and E is the number of environments.

## 3 Results

### 3.1 Analysis of variance

Analysis of variance for each trait varied differently. There were genotype significant differences for the studied traits in Serere, except husk cover in Bulindi, grain yield, husk cover and ear aspect in Ikulwe, days to anthesis and husk cover in Ngetta (Supplementary Table 1). Across environments, analysis of variance was significantly different (P < 0.01) for environment (E) for GY and all other measured traits. All genotypes (G) showed significant differences (P < 0.01) for GY and all other measured traits; and G × E interaction (GEI) showed significant difference (P < 0.05) for GY, AD (Table 2).

**Table 2:** Mean squares from ANOVA for grain yield agronomic traits and weevil resistance components, variance decomposition, and heritability of 58 testcross hybrids and 2 checks across 4 environments in Uganda (2016).

Source	df	Mean square				
		Grain yield (GY)	Days to anthesis (AD)	Grain texture (Tex)	Husk cover (HC)	Ear aspect (EA)
Environment (E)	3	104.22***	177.14***	5.191***	4109***	73.61***
Genotype (G)	59	4.43***	15.6***	2.759***	1698***	0.48***
GE	177	1.19*	6.93*	0.462	514	0.27
Residual	240	0.85	5.1	0.471	651	0.25

Genotypic variance		0.72	0.94	109.20	109.20	0.03
Environment variance		0.16	1.17	0.00	0.00	0.03
G × E variance		0.65	1.48	8.68	8.68	0.59
Residual variance		0.64	4.43	527.34	527.34	0.25
Heritability		0.82	0.53	0.84	0.62	0.47

		Grain weight loss (g)	Number of damaged kernels	Weevil mortality	Number of exit holes	Weight of powder (g)
Environment (E)	3	1746.7***	103,020***	53.7	203,773***	7.707
Genotype (G)	59	473.6***	16,954***	52.51**	35,259***	8.708
GE	177	159.1*	5652	35.71	11,136	8.316
Residual	240	125.4	4827	30.83	10,073	8.278

Genotypic variance		178.83	1894.99	4.32	3535.96	0.02
Environment variance		135.43	1237.98	8.56	2119.12	0.24
G × E variance		79.04	1294.95	0.20	2268.76	0.06
Residual variance		452.66	4819.02	40.00	8558.20	0.60
Heritability		0.60	0.58	0.20	0.59	0.06

### 3.2 Genotype performance at individual and across environments

Grain-yield of testcross hybrids varied in different environments. Lowest mean yield of 1.4 t/ha was obtained at Ikulwe, and the highest mean yield of 3.5 t/ha was recorded at Bulindi (Table 3). The best performing hybrid at Ngetta, Ikulwe, Serere, and Bulindi was 36%, 30%, 20%, and 14% above the best check hybrid, respectively. In ranking the environment in terms of grain yield potential: Bulindi > Serere > Ngetta > Ikulwe; and as in terms of heritability, they had a similar pattern as grain yield (Table 3). Average GY for top 15 test hybrids and the highest yielding hybrid over the checks was higher at Ngetta (15%–26%) and lowest (1%–3%) at Bulindi (Table 3).

**Table 3:** Mean performance of top 15 high-yielding testcross hybrids and commercial checks. Entries common under different environments are bolded and underlined.

Gen. No.	Cross	Environment													
		Ngetta		Serere				Bulindi				Ikulwe			
		GY	AD	Gen. No.	Entry	GY	AD	Gen. No.	Entry	GY	AD	Gen. No.	Entry	GY	AD
		(t ha^−1^)	(days)			(t ha^−1^)	(days)			(t ha^−1^)	(days)			(t ha^−1^)	(days)
G39	L2/T2	3.4	62.4	G23	L23/T1	5.0	63.6	G53	**L46/T2**	6.2	64.4	G39	L2/T2	3.4	65.5
G52	L45/T2	3.3	62.0	G24	L24/T1	4.8	66.1	G52	L45/T2	5.7	61.3	G9	L9/T1	2.9	64.0
G49	L42/T2	3.0	65.8	G28	L28/T1	4.6	66.5	G9	L9/T1	5.6	63.0	G43	L39/T2	2.6	67.0
G28	L28/T1	2.9	65.2	G39	L2/T2	4.5	63.9	G24	L24/T1	5.4	67.0	G42	L6/T2	2.4	66.0
G5	L5/T1	2.9	62.5	G43	L39/T2	4.4	64.0	G22	L22/T1	5.3	63.0	G53	**L46/T2**	2.4	66.5
G53	**L46/T2**	2.8	64.2	G22	L22/T1	4.3	64.9	G15	L15/T1	5.2	63.1	G20	L20/T1	2.4	67.5
G42	L6/T2	2.7	60.8	G27	L27/T1	4.2	63.6	G23	L23/T1	5.1	65.5	G55	L48/T2	2.2	65.0
G38	**L38/T2**	2.5	61.0	G52	L45/T2	4.2	62.6	G20	L20/T1	5.0	80.5	G23	L23/T1	2.1	67.0
G36	L36/TI	2.5	63.8	G29	L29/T1	4.1	63.9	G38	**L38/T2**	4.8	62.6	G36	L36/TI	2.1	64.5
G4	L4/T1	2.4	63.6	G9	L9/T1	4.0	62.5	G54	L47/T2	4.7	65.0	G38	**L38/T2**	1.9	67.5
G20	L20/T1	2.3	61.9	G55	L48/T2	3.9	63.5	G18	L18/T1	4.6	62.6	G8	L8/T1	1.9	66.0
G12	L12/T1	2.3	62.2	G38	**L38/T2**	3.9	62.9	G28	L28/T1	4.5	66.1	G33	L33/T1	1.8	64.0
G22	L22/T1	2.3	62.8	G53	**L46/T2**	3.9	65.0	G27	L27/T1	4.4	62.5	G57	L50/T2	1.8	66.5
G21	L21/T1	2.2	60.3	G50	L43/T2	3.7	66.6	G42	L6/T2	4.3	62.1	G17	L17/T1	1.7	66.0
G58	L51/T2	2.2	63.4	G8	L8/T1	3.7	63.4	G48	L41/T2	4.2	61.2	G25	L25/T1	1.7	66.0
G59	Commercial Check 1	2.5	61.4	G59	Check 1	3.7	66.1	G59	Commercial Check 1	6.1	65.8	G59	Commercial Check k 1	1.2	64.0
G60	Check 2	0.7	67.1	G60	Commercial Check 2	3.0	63.5	G60	Commercial Check k 2	3.3	62.2	G60	Commercial Check 2	2.5	67.0

	Grand Mean	1.8	63.0			3.4	63.8			3.5	63.3			1.4	66.6
	LSD_0.05_	1.6	3.0			1.3	2.5			1.9	6.5			1.7	2.0
	Heritability	0.5	0.5			0.8	0.7			0.8	0.4			0.1	0.7
	Mean of Top 15 hybrid	2.6				4.2				5.0				2.2	
	Mean of Checks	1.6				3.4				4.7				1.9	

	Advantage of top 15 hybrids over checks	26%				11%				3%				9%	
	Advantage of best hybrid over mean of checks	36%				20%				14%				30%	
	Advantage of best hybrid over the best check	15%				15%				1%				15%	

GY = Grain yield; AD = Days to anthesis.

Average GY of trials was 2.3 t/ha across the four environments (Table 4). The top 15 testcross hybrids gave 5.9% yield advantage over the best check and 13.5% yield advantage over the mean of the check. The best hybrid G39 (L2/T2) out yielded the best check (Check 1) by 11.4% across four environments. All genotypes had significant differences in maturity, with AD ranging from 61 to 69 days (Table 4).

**Table 4:** Mean performance of 15 high-yielding testcross hybrids and commercial checks across four environments in Uganda (2016). Entries that are consistent in individual environment and across environments are bolded and underlined.

Genotype No.	Cross	Grain yield	Days to anthesis	Grain texture	Husk cover	Ear aspect
		(t ha^−1^)	(days)	(1–5)	(%)	(1–5)
G39	**L2/T2**	3.9	63.7	3.3	15.7	2.6
G53	**L46/T2**	3.8	65.0	1.8	8.8	2.4
G23	**L23/T1**	3.6	64.8	2.1	10.2	2.3
G52	**L45/T2**	3.6	63.0	2.1	12.6	2.5
G9	**L9/T1**	3.5	63.2	2.3	14.6	2.5
G24	L24/T1	3.4	66.6	2.3	13.9	2.8
G20	L20/T1	3.4	69.2	2.5	9.0	2.2
G28	L28/T1	3.4	66.5	2.5	15.4	2.6
G22	L22/T1	3.3	64.4	2.1	15.6	2.5
G38	L38/T2	3.3	63.6	2.8	14.8	2.4
G43	L39/T2	3.1	64.1	2.8	12.3	2.6
G27	L27/T1	3.0	63.9	3.2	19.4	2.7
G42	L6/T2	3.0	63.0	2.7	41.0	2.8
G29	L29/T1	3.0	62.9	4.0	16.2	2.7
G36	L36/TI	2.9	64.4	3.6	14.2	2.9
G59	Check 1	3.5	64.9	2.1	7.8	2.7
G60	Check 2	2.5	63.8	2.0	11.2	2.9

	Min	0.3	61.3	1.6	7.8	2.5
	Max	3.9	69.2	4.0	74.9	3.5
	Grand Mean	2.3	64.2	2.7	22.7	2.8
	LSD_0.05_	0.9	1.9	0.7	24.1	0.4
	Mean of top 15 hybrid	3.3				
	Mean of checks	3.0				

	Advantage of top 15 hybrids over checks	5.9%				
	Advantage of best hybrid over mean of checks	13.5%				
	Advantage of best hybrid over the best check	5.7%				

### 3.3 Genetic variance and heritability for grain yield and its components

Overall, the effect of environment on grain yield explained 31.8% of total variance while genotype contributed about a third (35.4.0%), and GE contributed less than 4.7% of the total variation. Therefore, environment highly influenced the performance of different germplasm (Table 2). Most of the other traits followed similar a trend as grain yield. However, for grain texture, genotype effect explained 37.4%, and environment only 4.9%, and there was no contribution of GEI to the total variance. Heritability estimates among the different traits ranged from 0.47 to 0.84 across environments. With, 0.82 for GY, 0.53 for AD, 0.84 for TEX, 0.62 for HC and 0.47 for EA across environments (Table 2).

### 3.4 Response to weevil infestation

Analysis of variance for each weevil resistance trait varied differently. There were no significant differences among genotypes for the entire traits in Ngetta, weevil mortality and weight of powder from samples from Bulindi and Serere (Supplementary Table 1). Analysis of variance and performance of hybrids across environments showed significant differences (P < 0.01) in environment (E) for all other measured traits, except weevil mortality and weight of powder. All genotypes (G) showed significant differences (P < 0.01) for all measured traits except weight of powder; and G × E interaction (GEI) showed significant difference (P < 0.05) for only Grain weight loss (Table 2).

#### 3.4.1 Genotype performance at individual and across environments

The count of dead maize weevils (weevil mortality) during oviposition did not differ significantly among genotypes (Table 2, Table 5). Both number of damaged grain and grain weight loss were highly significant (*P* < 0.001) among genotypes. There were significant (*P* < 0.001) differences among genotypes for number of weevil exit holes, but there were no significant differences among genotypes for weight of the powder (Table 5). The mean number of dead maize weevils was 3.6. The lowest mortality of 1.1 was recorded for genotype G54 (L47/T2) and commercial check 2; while the highest (10.9) was observed on genotype G56 (L49/T2) (Table 5). The number of dead maize weevils on genotypes G24 (L24/T1), G25 (L25/T1) and G23 (L23/T1) were comparable with commercial check 1 (3.8–6.1).

**Table 5:** Means grain weight loss and number of damaged kernels, weevil mortality and other weevil resistance parameters of the 15 high-yielding testcross hybrids and commercial checks across four environments.

Entry	Cross	Grain weight loss (g)	Number of damaged kernels	Weevil mortality	Number of exit holes	Weight of powder (g)
G58	L51/T2	2.7^a^	21.8^a^	2.0^abc^	27.0^a^	0.1^a^
G16	L16/T1	2.9^a^	30.4^abc^	1.9^abc^	40.6^ab^	0.2^a^
G55	L48/T2	3.9^ab^	28.4^ab^	2.6^abcd^	40.4^ab^	0.3^a^
G24	L24/T1	4.0^ab^	32.8^abcd^	4.5^abcde^	49.4^abcde^	0.3^a^
G49	L42/T2	4.4^ab^	41.0^abcdef^	2.6^abcd^	53.3^abcdefg^	0.3^a^
G52	L45/T2	4.8^abc^	41.4^abcdef^	2.4^abcd^	48.0^abcd^	0.3^a^
G28	L28/T1	4.9^abcd^	59.0^bcdefghij^	3.5^abcd^	63.1^abcdefghij^	0.4^a^
G25	L25/T1	5.2^abcd^	37.6^abcdef^	5.5^abcde^	54.8^abcdefg^	0.4^a^
G36	L36/TI	5.2^abcd^	44.0^abcdefgh^	2.8^abcd^	60.1^abcdefgh^	0.5^a^
G54	L47/T2	5.5^abcd^	40.8^abcdef^	1.1^ab^	66.6^abcdefghijk^	0.3^a^
G23	L23/T1	5.5^abcd^	48.0^abcdefgh^	3.8^abcde^	72.1^abcdefghijk^	0.4^a^
G56	L49/T2	5.5^abcd^	52.0^abcdefghi^	10.9^f^	73.3^abcdefghijk^	0.4^a^
G12	L12/T1	5.5^abcd^	50.3^abcdefgh^	2.4^abcd^	63.5^abcdefghij^	0.4^a^
G5	L5/T1	5.8^abcd^	52.3^abcdefghi^	3.3^abcd^	71.0^abcdefghijk^	0.5^a^
G40	L3/T2	5.9^abcd^	62.0^bcdefghij^	2.5^abcd^	84.8^bcdefghijklm^	0.4^a^
G59	Check 1	5.2^abcd^	42.6^abcdef^	6.1^bcdef^	61.4^abcdefghi^	0.3^a^
G60	Check 2	9.4^abcdef^	35.4^abcde^	1.1^ab^	42.0^abc^	0.2^a^

	Mean	10.9	56.2	3.6	79.1	0.7
	*P*	***	***	NS	***	NS
	SE	4.5	12.3		17	
	LSD_0.05_	12.1	34.1		47.3	
	Mean of Top 15 hybrid	4.8	42.8	3.4	57.9	0.3
	Mean of Checks	7.3	39.0	3.6	51.7	0.3

^NS^ not significant, *, **, *** denotes significant at *P* < 0.05, 0.01, and 0.001 respectively. Means with the same letters in the same column are not significantly different according to Fisher's Protected LSD.

The mean number of damaged kernels recorded was 56.2, with genotype G58 (L51/T2) recording less damaged kernels compared to both checks. Hybrids G58 (L51/T2) and G16 (L16/T1) had the least grain weight reduction of 2.7 and 2.9 respectively, which was significantly lower than loss suffered by the commercial check 2 (Table 5). The mean of the number of exit holes was 79.1 with the lowest value noted for genotype G58 (L51/T2), while the highest was observed on genotype G40 (L3/T2) (Table 5) among the top best 15 genotypes.

#### 3.4.2 Genetic variance and heritability for weevil resistance traits

Overall, on kernel damage which we used as a proxy for resistance, environmental effect explained 14.8% of the total variance while genotype explained about a third (18.1%), and GE contributed less than 12.3% of the total variation. Therefore, environment did not influence a lot on the performance of the different germplasm rather than the residual (Table 2). Most of the other traits followed a similar trend with residual contributing a lot to the total variation. Heritability estimates among the different traits ranged from 0.06 to 0.60 across environments, with 0.58 for kernel damage, 0.22 for weevil mortality for AD, 0.59 for number of exit holes, 0.60 for grain weight loss, and 0.06 for weight of powder across environments (Table 2).

### 3.5 Correlations between agronomic traits and parameters of maize weevil resistance

Pearson correlation coefficients between agronomic traits and different maize weevil resistance traits varied in magnitude. Weight loss was positive and significantly correlated with number of damaged kernels (*r* = 0.53; *P* < 0.001), number of exit holes (*r* = 0.54; *P* < 0.001), and ear aspect (*r* = 0.29; *P* < 0.05) (Table 6). Maize weevil mortality was positively and significantly correlated with grain texture (*r* = 0.26; P < 0.05). Further, there was a strong positive and highly significantly correlation between number of damaged kernels and number of exit holes (*r* = 0.96; *P* < 0.001).

**Table 6:** Pearson correlation coefficients among maize weevil resistance parameters for 15 high-yielding testcross hybrids and commercial checks across four environments.

	Grain weight loss (g)	Number of damaged kernels	Weevil mortality	Number of exit holes	Weight of powder	Grain Texture
Grain weight loss (g)						
Number of damaged kernels	0.53***					
Weevil mortality	0.16	0.22				
Number of exit holes	0.54***	0.96***	0.22			
Weight of powder	0.12	0.20	−0.03	0.19		
Grain texture	0.20	0.16	0.26*	0.11	0.12	
Ear aspect	0.29*	0.20	0.04	0.21	0.24	0.39**

*, **, *** denotes significant at P < 0.05, 0.01, a 0.001 respectively.

## 4 Discussion

The study examined agronomic performance and maize weevil resistance among early generation testcross hybrids. Results showed significant variation in yield in different environments that might possibly be ascribed to differences in factors of climate such as rainfall, soil fertility status and type, and temperature at the different environments used in the study. Several authors have reported variations caused by various climatic factors (Butron et al., 2002; Gorman et al., 1989; Igartua, 1995; Kays and Nottingham, 2007). Presence of significant variation among genotypes suggests that selection could be effectively made in this set of germplasm. High grain yield combined with stable performance across sites, and satisfactory performance levels for key adaptive traits like disease resistance are important criteria for choosing genotypes to advance through the stage-gate process. The F_2:3_ lines used in this study potentially possess useful variation that can be exploited in breeding of high yielding maize hybrids for diverse agro-ecologies in Uganda. Inbred line parents of the best hybrids in terms of grain yield across environments (L28, L23, L2) are some of the lines with good yield potential that may be further used in the breeding program. Reports have deduced that early generation testing offers a highly promising tool for the identification of required like quality good/high yielding genotypes at early growth stages, and casts off the poor combiners (Ali et al., 2013; Dari et al., 2010).

The implication of significant genotype × environment interaction implies that there is differential hybrid performance across variable conditions. Similar observations were reported by several authors (Beyene et al., 2013; Ertiro et al., 2017; Sserumaga et al., 2018, 2016) who studied about adaptability and performance of maize hybrids under different stress conditions in eastern Africa.

Results revealed variation in magnitude of variances for GY with environment that accounted for the largest proportion followed by genotype, and genotype × environment interactions. The results are similar to those reported by Setimela et al. (2010, 2007), Beyene et al. (2011), Makumbi et al. (2015) and Sserumaga et al. (2018, 2016). The large environmental variance indicates that the testing sites were highly variable from one environment to another. For example, Ikulwe was ranked last in terms of grain yield although it had similar rainfall (1338–1345 mm) pattern as Bulindi, which ranked highest in terms of grain yield. This could be as a result of the presence of plinthite (hardpan; Plinthosols) in the subsoil that are characterized by being highly weathered soil and restrict root growth and utilization of moisture and nutrients from the subsoil by the maize plants (Beinroth et al., 1996; Eswaran et al., 1990; Eze et al., 2014; Staff, 2010). Similar reports by Butron et al. (2002) suggested that G × E affects yield mainly through the environmental yield-limiting factors including rainfall, relative humidity, mean minimum temperature and soil nutrients. The presence of large G × E obfuscates selection decision, the performance of elite genotypes befit conditional on the particular environment where they are planted (Rattey and Kimbeng, 2001; Zhou et al., 2012). Like yield, which is a quantitatively inherited trait, the genotype values and their relative rankings change significantly as per the environment (Kang, 2002), hence confounds the determination of true genetic value of the potential varieties (Haruna et al., 2017; Kimbeng et al., 2009; Zhou et al., 2012). So, when G × E is significant, breeders need to precisely sample the target environmental conditions where these varieties will be produced after their release. Hence need to set trials at several environments (Haruna et al., 2017; Kimbeng et al., 2009; Zhou et al., 2012).

According to Robinson (1963), broad-sense heritability is an estimate of the narrow-sense heritability upper limit. High heritability and genetic variation are ideal conditions for effective germplasm selection (Falconer and Mackay, 1996; Sleper and Poehlman, 2006). In the current study, we reported modest broad-sense heritability (0.61–0.64) for days to anthesis (AD), ear aspect (EA) and husk cover (HC), suggesting that actual heritability estimates might be lower (Falconer and Mackay, 1996), which could lead to low genetic gain from selection for these traits in this germplasm. Conversely, broad sense heritability estimate for grain yield was 0.87, signifying that genuine heritability estimates might be high (Falconer and Mackay, 1996), which could lead to higher genetic gain from selection. These results concur with those reported earlier by Sserumaga et al. (2016) while evaluating doubled haploids hybrids in East Africa, but are in contrast with the results reported by Kanyamasoro et al. (2012) that showed low heritability for grain yield. Heritability estimates for grain yield generally vary with germplasm under test.

Resistance to maize weevil was measured using five parameters namely grain weight loss, number of damaged kernels, weevil mortality, number of exit holes, and weight of powder. Assessment of grain weight loss among hybrids revealed significant variation suggesting presence of genotypic differences in maize weevil resistance in this germplasm. Similar findings were reported by Tefera et al. (2011), Masasa et al. (2013), Zunjare et al. (2014), Derera et al. (2014) and Kasozi et al. (2015) in studies with different types of maize germplasm. The large variation for grain weight loss for these germplasm means that the selection could be an effective strategy for maize weevil resistance improvement. All hybrids succumbed to grain weight losses when exposed to the maize weevil, suggesting that resistance was partial. This is in line with previous scrutiny of additive gene action for weevil resistance in maize (Derera et al., 2014). Grain weight loss variations could be attributed to intrinsic differences in physical kernel traits among the genotypes evaluated (Masasa et al., 2013).

Maize weevil mortality was not significantly different among the genotypes. This is consistent with results by Dhliwayo et al. (2005) and Kasozi et al. (2015) but in contrast to findings by Nhamucho et al. (2017) who reported significant differences in maize weevil mortality among Mozambican local and improved maize genotypes. Mortality maybe attributed to presence of a fluorescent pericarp with high concentration of hydroxycinnamic acid (Serratos et al., 1987). These phenolic compounds bound to the arabinoxylans within the cell wall which makes it difficult for the maize weevil to degrade the pericarp (Masasa et al., 2013; Serratos et al., 1987). Derera et al. (2010) suggested that weevil mortality could be used to assess resistance to maize weevil but results of this study indicate that this parameter may not be a good indicator of resistance.

Resistance to maize weevil as measured by number of damaged kernels showed highly significant variation among the hybrids. Siwale et al., 2010, Abebe et al. (2009), Masasa et al. (2013) and Kasozi et al. (2015) also reported significant variation among hybrids. The weight of powder produced after eating by the maize weevil did not differ significantly among the hybrids, which suggested successful infestation and feeding by the insects. Weight of powder produced may not be a good measure of weevil resistance. This is in contrast with previous studies that reported significant variation in weight of powder among different types of maize varieties when infested with the maize weevil (Mwololo et al., 2012; Suleiman et al., 2015; Tefera et al., 2011). It is important to note that the flour produced during the insects’ feeding consists of insect eggs, excreta and exuviae that are unfit for both livestock and human consumption (Tefera et al., 2011).

Maize weevil resistance measured by different parameters was weakly correlated with grain texture and ear aspect, which suggested that these two traits are not reliable indicators of weevil resistance. Other studies also reported low correlation between grain texture and weevil resistance (Firoz et al., 2007; Schoonhoven et al., 1972; Singh and McCain, 1963). Depending on the population under study, many mechanisms of resistance and their importance vary (Derera et al., 2014). Other studies reported that the grain resistance to storage insect attack was attributed to a number of factors that are genetic, physical or environmental including antibiosis, husk protection, kernel size and pericarp surface texture, kernel hardness, starchy amylose content, antifeedant compounds such as phenolics, presence of toxic alkaloids, and moisture content and grain temperature (Abebe et al., 2009; Goftishu and Belete, 2014; Keba and Sori, 2013; Suleiman et al., 2015). These factors may act alone or in combinations to reduce effect of stored grain insect damage (Goftishu and Belete, 2014). The number of exit holes was highly and significantly correlated with number of damaged kernels in this study, which suggested that one of these parameters is sufficient for weevil resistance assessment in a breeding program.

## 5 Conclusions

The study used early generation inbred lines under development in maize breeding program of NARO, Uganda and, crossed with common testers, we identified hybrids with higher grain yield and weevil resistance than best commercial check. Selection of the best line-tester combination identified in the study (e.g. L2/T2, L46/T2, L23/T1, L45/T2, L9/T1, L51/T2, L16/T1, L48/T2, L24/T1, L42/T2 and L45/T2) with higher mean yield and weevil resistant across environments would contribute to the development of lines that will form hybrids with productivity for smallholder farmers in SSA. Knowledge about correlation between traits can be utilized in decisions regarding indirect selection when breeding for stress tolerance and ultimately when designing a breeding strategy. The new eleven identified lines (L2, L9, L16, L23, L24, L38, L42, L45, L46, L48, and L51) that have promising grain yield and other secondary traits, could be advanced as candidate inbred lines for the development of new inbred lines. Of these, three new lines (L48, L23, and L45) were identified to have resistance to weevil. Results suggested that good lines can be identified at early stage of inbred line development that could be used to develop lines and hybrids with multiple stress-tolerances.

## Declaration of competing interest

The authors declare that there is no conflict of interest.

## References

[bib1] Abass A.B., Ndunguru G., Mamiro P., Alenkhe B., Mlingi N., Bekunda M. (2014). Post-harvest food losses in a maize-based farming system of semi-arid savannah area of Tanzania. J. Stored Prod. Res..

[bib2] Abebe F., Tadele T., Stephen M., Yoseph B., Stefan V. (2009). Resistance of maize varieties to the maize weevil *Sitophilus zeamais* (Motsch.) (Coleoptera: Curculionidae). Afr. J. Biotechnol..

[bib3] Adebayo O.J., Omoloye A. (2012). Rearing the maize weevil, *Sitophilus zeamais*, on an artificial maize–cassava diet. J. Insect Sci..

[bib4] Ali F., Zahid K.R., Shah F., Gul R., Pan Q., Mustafa G., Jamal Y., Khan H., Ullah H. (2013). Heterosis and early generation testing is a pivotal method for production of hybrid. Aust. J. Crop. Sci..

[bib5] Alvarado G., Rodríguez F.M., Pacheco A., Burgueño J., Crossa J., Vargas M., Pérez-Rodríguez P., Lopez-Cruz M.A. (2020). META-R: a software to analyze data from multi-environment plant breeding trials. Crop J.

[bib6] Asawalam E.F., Hassanali A. (2006). Constituents of the essential oil of Vernonia amygdalina as maize weevil protectants. Trop. Subtrop. Agroecosystems.

[bib7] Beinroth F.H., Eswaran H., Palmieri F., Reich P.F. (1996). Properties, Classification and Management of Oxisols.

[bib8] Beyene Yoseph, Mugo Stephen, Gakunga John, Karaya Haron, Mutinda Charles, Tefera Tadele, Njoka Stephen, Chepkesis Dorcas, Shuma Jackson M., Tende Regina (2011). Combining ability of maize (*Zea mays* L.) inbred lines resistant to stem borers. Afr. J. Biotechnol..

[bib9] Beyene Y., Mugo S., Semagn K., Asea G., Trevisan W., Tarekegne A., Tefera T., Gethi J., Kiula B., Gakunga J. (2013). Genetic distance among doubled haploid maize lines and their testcross performance under drought stress and non-stress conditions. Euphytica.

[bib10] Beyene Y., Semagn K., Mugo S., Prasanna B.M., Tarekegne A., Gakunga J., Sehabiague P., Meisel B., Oikeh S.O., Olsen M., Crossa J. (2016). Performance and grain yield stability of maize populations developed using marker-assisted recurrent selection and pedigree selection procedures. Euphytica.

[bib11] Boxall R.A. (1986). A Critical Review of the Methodology for Assessing Farm-Level Grain Losses after Harvest.

[bib12] Butron A., Widstrom N., Snook M., Wiseman B. (2002). Recurrent selection for corn earworm (Lepidoptera: noctuidae) resistance in three closely related corn southern synthetics. J. Econ. Entomol..

[bib13] Dari S., Pixley K.V., Setimela P. (2010). Resistance of early generation maize inbred lines and their hybrids to maize weevil [(Motschulsky)]. Crop Sci..

[bib14] Derera J., Pixley K.V., Giga D.P. (2010). Appraisal of protocol for the rapid screening of maize genotypes for maize weevil resistance. Afr. Entomol..

[bib15] Derera J., Pixley K.V., Giga D.P., Makanda I. (2014). Resistance of maize to the maize weevil: III. Grain weight loss assessment and implications for breeding. J. Stored Prod. Res..

[bib16] Dhliwayo T., Pixley K.V., Kazembe V. (2005). Combining ability for resistance to maize weevil among 14 southern African maize inbred lines. Crop Sci..

[bib17] Dobie P. (1974). The laboratory assessment of the inherent susceptibility of maize varieties to post-harvest infestation by *Sitophilus zeamais* Motsch. (Coleoptera, Curculionidae). J. Stored Prod. Res..

[bib18] Ertiro B.T., Beyene Y., Das B., Mugo S., Olsen M., Oikeh S., Juma C., Labuschagne M., Prasanna B.M. (2017). Combining ability and testcross performance of drought-tolerant maize inbred lines under stress and non-stress environments in Kenya. Plant Breed..

[bib19] Eswaran H., De Coninck F., Varghese T. (1990). Role of plinthite and related forms in soil degradation. Advances in Soil Science.

[bib20] Eze P.N., Udeigwe T.K., Meadows M.E. (2014). Plinthite and its associated evolutionary forms in soils and landscapes: a review. Pedosphere.

[bib21] Falconer D., Mackay T. (1996). Introduction to Quantitative Genetics.

[bib22] FAOSTAT (2016). Food and Agricultural organization of the united nations (FAO), FAO statistical database. http://faostat3.fao.org/download/Q/QC/EFAOSTAT.

[bib23] Firoz H., Boddupalli P.M., Sharma R.K., Kumar P., Singh B.B. (2007). Evaluation of quality protein maize genotypes for resistance to stored grain weevil *Sitophilus oryzae* (Coleoptera: Curculionidae). Int. J. Trop. Insect Sci..

[bib24] Fischer R.A., Rebetzke G.J. (2018). Indirect selection for potential yield in early-generation, spaced plantings of wheat and other small-grain cereals: a review. Crop Pasture Sci..

[bib25] Goftishu M., Belete K. (2014). Susceptibility of sorghum varieties to the maize weevil *Sitophilus zeamais* Motschulsky (Coleoptera: Curculionidae). Afr. J. Agric. Res..

[bib26] Gorman D.P., Kang M.S., Milam M.R. (1989). Contribution of weather variables to genotype X environment interaction in grain sorghum. Plant Breed..

[bib28] Guedes R.N.C., Lima J.G., Santos J.P., Cruz C.D. (1995). Resistance to DDT and pyrethroids in Brazilian populations of *Sitophilus zeamais* Motsch. (Coleoptera: Curculionidae). J. Stored Prod. Res..

[bib29] Guthrie W.D., Lillehoj E.B., McMillian W.W., Barry D., Kwolek W.F., Franz A.O., Catalano E.A., Russel W.A., Widstrom N.W. (1981). Effect of hybrids with different levels of susceptibility to second-generation European corn borers on aflatoxin contamination in corn. J. Agric. Food Chem..

[bib30] Hallauer A.R., Carena M.J., Miranda Filho J.B. (2010). Quantitative Genetics in Maize Breeding.

[bib31] Haruna A., Adu G.B., Buah S.S., Kanton R.A.L., Kudzo A.I., Seidu A.M., Kwadwo O.-A. (2017). Analysis of genotype by environment interaction for grain yield of intermediate maturing drought tolerant top-cross maize hybrids under rain-fed conditions. Cogent Food Agric.

[bib32] Igartua E. (1995). Choice of selection environment for improving crop yields in saline areas. Theor. Appl. Genet..

[bib33] Kang M.S. (2002). Quantitative Genetics, Genomics, and Plant Breeding.

[bib34] Kanyamasoro M.G., Karungi J., Asea G., Gibson P. (2012). Determination of the heterotic groups of maize inbred lines and the inheritance of their resistance to the maize weevil. Afr. Crop Sci. J..

[bib35] Kasozi L.C., Derera J., Tongoona P. (2015). Response of maize population “Longe 5” to two cycles of modified S1 recurrent selection for resistance to maize weevil. Euphytica.

[bib36] Kays S., Nottingham S. (2007). Biology and Chemistry of Jerusalem Artichoke: Helianthus Tuberosus L..

[bib37] Keba T., Sori W. (2013). Differential resistance of maize varieties to maize weevil *(Sitophilus zeamais* Motschulsky) (Coleoptera: Curculionidae) under laboratory conditions. J. Entomol..

[bib38] Kimbeng C.A., Zhou M.M., Da Silva J.A. (2009). Genotype x environment interactions and resource allocation in sugarcane yield trials in the Rio Grande valley region of Texas. J. Am. Soc. Sugar Cane Technol..

[bib39] Kljajić P., Perić I. (2006). Susceptibility to contact insecticides of granary weevil *Sitophilus granarius* (L.) (Coleoptera: Curculionidae) originating from different locations in the former Yugoslavia. J. Stored Prod. Res..

[bib40] Makumbi D., Diallo A., Kanampiu K., Mugo S., Karaya H. (2015). Agronomic performance and genotype x environment interaction of herbicide-resistant maize varieties in eastern *Africa*. Crop Sci..

[bib41] Masasa R.T., Setimela P.S., Chiteka Z.A. (2013). Evaluation of open pollinated varieties of maize for resistance to the maize weevil in a controlled temperature and humidity laboratory in Zimbabwe. Euphytica.

[bib44] Mwololo J.K., Mugo S., Okori P., Tefera T., Otim M., Munyiri S.W. (2012). Sources of resistance to the maize weevil *Sitophilus zeamais* in tropical maize. J of Agri Science.

[bib45] Nhamucho E., Mugo S., Gohole L., Tefera T., Kinyua M., Mulima E. (2017). Resistance of selected Mozambican local and improved maize genotypes to maize weevil, *Sitophilus zeamais* (Motschulsky). J. Stored Prod. Res..

[bib46] OECD/FAO (2018). Agriculture in sub-saharan Africa: prospects and challenges for the next decade.

[bib47] Oliveira E.E., Guedes R.N.C., Tótola M.R., De Marco P. (2007). Competition between insecticide-susceptible and -resistant populations of the maize weevil, *Sitophilus zeamais*. Chemosphere.

[bib49] Patterson H.D., Williams E.R. (1976). A new class of resolvable incomplete block designs. Biometrika.

[bib50] Payne R.W., Murray D.A., Harding S.A., Baird D.B., Soutar D.M. (2012). GenStat for windows introduction. VSN Int. Hemel Hempstead.

[bib51] Pingali P.L. (2001). CIMMYT 1999–2000 World Maize Facts and Trends. Meeting World MaizeNeeds: Technological Opportunities and Priorities for the Public Sector. Mexico, D.F.: CIMMYT.

[bib52] Rattey A.R., Kimbeng C.A. (2001). Genotype by environment interactions and resource allocation in final stage selection trials in the Burdekin district.

[bib53] Ribeiro B.M., Guedes R.N.C., Oliveira E.E., Santos J.P. (2003). Insecticide resistance and synergism in Brazilian populations of *Sitophilus zeamais* (Coleoptera: Curculionidae). J. Stored Prod. Res..

[bib54] Robinson P., Hanson W.D., Robinson H.F. (1963). Heritability: a second look. Statistical Genetics and Plant Breeding.

[bib55] SAS (2011). SAS/STAT Users Guide 9.3.

[bib56] Schoonhoven A. Van, Horber E., Mills R.B., Wassom C.E. (1972). Resistance in corn kernels to the maize weevil, *Sitophilus zeamais* Motsch. Entomol Soc Amer N Cent Br Proc.

[bib57] Serratos A., Arnason J.T., Nozzolillo C., Lambert J.D.H., Philogène B.J.R., Fulcher G., Davidson K., Peacock L., Atkinson J., Morand P. (1987). Factors contributing to resistance of exotic maize populations to maize weevil, *Sitophilus zeamais*. J. Chem. Ecol..

[bib58] Setimela P.S., Crossa J., Bänziger M. (2010). Targeting of early to intermediate maize hybrids for yield performance and yield stability using SREG model. S. Afr. J. Plant Soil.

[bib59] Setimela P.S., Vivek B., Bänziger M., Crossa J., Maideni F. (2007). Evaluation of early to medium maturing open pollinated maize varieties in SADC region using GGE biplot based on the SREG model. Field Crop. Res..

[bib60] Singh D., McCain F. (1963). Relationship of some nutritional properties of the corn kernel to weevil infestation. Crop Sci..

[bib61] Siwale J., Mbata K., Microbert J., Lungu D. (2010). Comparative resistance of improved maize genotypes and landraces to maize weevil. Afr. Crop Sci. J..

[bib62] Sleper D., Poehlman J. (2006). Breeding Field Crops.

[bib63] Sserumaga J.P., Beyene Y., Pillay K., Kullaya A., Oikeh S.O., Mugo S., Machida L., Ngolinda I., Asea G., Ringo J., Otim M., Abalo G., Kiula B. (2018). Grain-yield stability among tropical maize hybrids derived from doubled-haploid inbred lines under random drought stress and optimum moisture conditions. Crop Pasture Sci..

[bib64] Sserumaga J.P., Oikeh S.O., Mugo S., Asea G., Otim M., Beyene Y., Abalo G., Kikafunda J. (2016). Genotype by environment interactions and agronomic performance of doubled haploids testcross maize *(Zea mays* L.) hybrids. Euphytica.

[bib65] Staff S. (2010). Keys to Soil Taxonomy.

[bib66] Suleiman R., Rosentrater K.A., Bern C.J. (2015). Evaluation of maize weevils *Sitophilus zeamais* Motschulsky infestation on seven varieties of maize. J. Stored Prod. Res..

[bib67] Tefera T., Mugo S., Likhayo P., Beyene Y. (2011). Resistance of three-way cross experimental maize hybrids to post-harvest insect pests, the larger grain borer (*Prostephanus truncatus*) and maize weevil (*Sitophilus zeamais*). *Int. J. Trop. Insect* Sci..

[bib68] Vivek B.S., Odongo O., Njuguna J., Imanywoha J., Bigirwa G., Pixley K. (2010). Diallel analysis of grain yield and resistance to seven diseases of 12 African maize (*Zea mays* L.) inbred lines. Euphytica.

[bib69] Yamada Y. (1962). Genotype by environment interaction and genetic correlation of the same trait under different environments. Jpn. J. Genet..

[bib70] Zhou M., Joshi S.V., Maritz T. (2012). Trends and implications of genotype by environment interaction in South African sugarcane breeding. J. Crop Improv..

[bib71] Zunjare R., Hossain F., Thirunavukkarasu N., Muthusamy V., Jha S.K., Kumar P., Gupta H.S. (2014). Evaluation of specialty corn inbreds for responses to stored grain weevil (*Sitophilus oryzae* L.) infestation. Indian J. Genet. Plant Breed..

