# Supplementary material for: Evaluation of early-generation tropical maize testcrosses for grain-yield potential and weevil (Sitophilus zeamais Motschulsky) resistance
Source: Crop Prot. 2021 Jan;139:105384. doi: 10.1016/j.cropro.2020.105384 (PMC7649906; doi:10.1016/j.cropro.2020.105384)
Supplement: Multimedia component 1 [file mmc1.docx]

Table 1: List of inbred lines used to form segregating population

| No. | Name | Pedigree | Source | Attribute |
| --- | --- | --- | --- | --- |
| 1 | NML85 | [KILIMA(ST94)-S5:115/[M37W/ZM607#BF37SR…]]-B-B-1-3-#-B | NARO | Drought tolerant, GLS, TLB and MSV resistant |
| 2 | NML88 | [KILIMA(ST94)-S5:115/[M37W/ZM607#BF37SR…]]-B-B-1-6-#-B | NARO | Drought tolerant, GLS, TLB and MSV resistant |
| 3 | NML97 | [[EV7992#/EV8449-SR]C1F2-334-1(OSU9i)-8-6(I)-X-X-1-B-B/CML206]-B-B-2-1-#-B | NARO | Drought tolerant, GLS, TLB and MSV resistant |
| 4 | CKDHL0165 | (La Posta Seq C7-F96-1-2-1-1-B-B-B/CML444/CML444) DH-104-B-B-B | CIMMYT | Drought tolerant, GLS, TLB and MSV resistant |
| 5 | CKDHL0216 | (La Posta Seq C7-F71-1-2-1-2-B-B-B/CML312SR = MAS[MSR/312]-117-2-2-1-2-B*4-B-B-B-B/CML312SR) DH-5-B-B-B | CIMMYT | Drought tolerant, GLS, TLB and MSV resistant |
| 6 | CKDHL0221 | (La Posta Seq C7-F71-1-2-1-2-B-B-B/CML312SR = MAS[MSR/312]-117-2-2-1-2-B*4-B-B-B-B/CML312SR) DH-10-B-B-B | CIMMYT | Drought tolerant, GLS, TLB and MSV resistant |
| 7 | CKDHL0227 | (La Posta Seq C7-F71-1-2-1-2-B-B-B/CML312SR = MAS[MSR/312]-117-2-2-1-2-B*4-B-B-B-B/CML312SR) DH-18-B-B-B | CIMMYT | Drought tolerant, GLS, TLB and MSV resistant |
| 8 | CKDHL0277 | (La Posta Seq C7-F71-1-2-1-2-B-B-B/CML312SR = MAS[MSR/312]-117-2-2-1-2-B*4-B-B-B-B/CML312SR) DH-100-B-B-B | CIMMYT | Drought tolerant, GLS, TLB and MSV resistant |
| 9 | CKDHL0295 | (La Posta Seq C7-F71-1-2-1-2-B-B-B/CML395/CML395) DH-21-B-B-B | CIMMYT | Drought tolerant, GLS, TLB and MSV resistant |
| 10 | CKDHL0333 | (La Posta Seq C7-F71-1-2-1-2-B-B-B/CML395/CML395) DH-65-B-B-B | CIMMYT | Drought tolerant, GLS, TLB and MSV resistant |
| 11 | CKDHL0373 | (La Posta Seq C7-F71-1-2-1-2-B-B-B/CML395/CML395) DH-107-B-B-B | CIMMYT | Drought tolerant, GLS, TLB and MSV resistant |
| 12 | CKDHL0470 | (La Posta Seq C7-F71-1-2-1-2-B-B-B/CML444/CML444) DH-49-B-B-B | CIMMYT | Drought tolerant, GLS, TLB and MSV resistant |

Table 2: Mean squares from ANOVA for grain yield agronomic traits and weevil resistance components, variance decomposition, and heritability of 58 testcross hybrids and 2 checks across 4 environments in Uganda, 2016.

|  |  | Mean square | | | | |
| --- | --- | --- | --- | --- | --- | --- |
| Source | df | Grain yield (GY) | Days to anthesis (AD) | Grain texture (Tex) | Husk cover (HC) | Ear aspect (EA) |
|  |  |  |  |  |  |  |
| Environment (E) | 3 | 104.22*** | 177.14*** | 5.191*** | 4109*** | 73.61*** |
| Genotype (G) | 59 | 4.43*** | 15.6*** | 2.759*** | 1698*** | 0.48*** |
| GE | 177 | 1.19* | 6.93* | 0.462 | 514 | 0.27 |
| Residual | 240 | 0.85 | 5.1 | 0.471 | 651 | 0.25 |
|  |  |  |  |  |  |  |
| Genotypic variance |  | 0.72 | 0.94 | 109.20 | 109.20 | 0.03 |
| Environment variance |  | 0.16 | 1.17 | 0.00 | 0.00 | 0.03 |
| G × E variance |  | 0.65 | 1.48 | 8.68 | 8.68 | 0.59 |
| Residual variance |  | 0.64 | 4.43 | 527.34 | 527.34 | 0.25 |
| Heritability |  | 0.82 | 0.53 | 0.84 | 0.62 | 0.47 |
|  |  |  |  |  |  |  |
|  |  | Grain weight loss (g) | Number of damaged kernels | Weevil mortality | Number of exit holes | Weight of powder (g) |
| Environment (E) | 3 | 1746.7*** | 103020*** | 53.7 | 203773*** | 7.707 |
| Genotype (G) | 59 | 473.6*** | 16954*** | 52.51** | 35259*** | 8.708 |
| GE | 177 | 159.1* | 5652 | 35.71 | 11136 | 8.316 |
| Residual | 240 | 125.4 | 4827 | 30.83 | 10073 | 8.278 |
|  |  |  |  |  |  |  |
| Genotypic variance |  | 178.83 | 1894.99 | 4.32 | 3535.96 | 0.02 |
| Environment variance |  | 135.43 | 1237.98 | 8.56 | 2119.12 | 0.24 |
| G × E variance |  | 79.04 | 1294.95 | 0.20 | 2268.76 | 0.06 |
| Residual variance |  | 452.66 | 4819.02 | 40.00 | 8558.20 | 0.60 |
| Heritability |  | 0.60 | 0.58 | 0.20 | 0.59 | 0.06 |

Table 3: Mean performance of top 15 high-yielding testcross hybrids and commercial checks. Entries common under different environments are bolded and underlined

|  |  | | Environment | | | | | | | | | | | | | | | | |
| --- | --- | --- | --- | --- | --- | --- | --- | --- | --- | --- | --- | --- | --- | --- | --- | --- | --- | --- | --- |
|  |  | | Ngetta | |  | Serere | | | |  | Bulindi | | | |  | Ikulwe | | | |
| Gen. No. | | Cross | GY | AD |  | Gen. No. | Entry | GY | AD |  | Gen. No. | Entry | GY | AD |  | Gen. No. | Entry | GY | AD |
|  | |  | (t ha^-1^) | (days) |  |  |  | (t ha^-1^) | (days) |  |  |  | (t ha^-1^) | (days) |  |  |  | (t ha^-1^) | (days) |
| G39 | | L2/T2 | 3.4 | 62.4 |  | G23 | L23/T1 | 5.0 | 63.6 |  | G53 | **L46/T2** | 6.2 | 64.4 |  | G39 | L2/T2 | 3.4 | 65.5 |
| G52 | | L45/T2 | 3.3 | 62.0 |  | G24 | L24/T1 | 4.8 | 66.1 |  | G52 | L45/T2 | 5.7 | 61.3 |  | G9 | L9/T1 | 2.9 | 64.0 |
| G49 | | L42/T2 | 3.0 | 65.8 |  | G28 | L28/T1 | 4.6 | 66.5 |  | G9 | L9/T1 | 5.6 | 63.0 |  | G43 | L39/T2 | 2.6 | 67.0 |
| G28 | | L28/T1 | 2.9 | 65.2 |  | G39 | L2/T2 | 4.5 | 63.9 |  | G24 | L24/T1 | 5.4 | 67.0 |  | G42 | L6/T2 | 2.4 | 66.0 |
| G5 | | L5/T1 | 2.9 | 62.5 |  | G43 | L39/T2 | 4.4 | 64.0 |  | G22 | L22/T1 | 5.3 | 63.0 |  | G53 | **L46/T2** | 2.4 | 66.5 |
| G53 | | **L46/T2** | 2.8 | 64.2 |  | G22 | L22/T1 | 4.3 | 64.9 |  | G15 | L15/T1 | 5.2 | 63.1 |  | G20 | L20/T1 | 2.4 | 67.5 |
| G42 | | L6/T2 | 2.7 | 60.8 |  | G27 | L27/T1 | 4.2 | 63.6 |  | G23 | L23/T1 | 5.1 | 65.5 |  | G55 | L48/T2 | 2.2 | 65.0 |
| G38 | | **L38/T2** | 2.5 | 61.0 |  | G52 | L45/T2 | 4.2 | 62.6 |  | G20 | L20/T1 | 5.0 | 80.5 |  | G23 | L23/T1 | 2.1 | 67.0 |
| G36 | | L36/TI | 2.5 | 63.8 |  | G29 | L29/T1 | 4.1 | 63.9 |  | G38 | **L38/T2** | 4.8 | 62.6 |  | G36 | L36/TI | 2.1 | 64.5 |
| G4 | | L4/T1 | 2.4 | 63.6 |  | G9 | L9/T1 | 4.0 | 62.5 |  | G54 | L47/T2 | 4.7 | 65.0 |  | G38 | **L38/T2** | 1.9 | 67.5 |
| G20 | | L20/T1 | 2.3 | 61.9 |  | G55 | L48/T2 | 3.9 | 63.5 |  | G18 | L18/T1 | 4.6 | 62.6 |  | G8 | L8/T1 | 1.9 | 66.0 |
| G12 | | L12/T1 | 2.3 | 62.2 |  | G38 | **L38/T2** | 3.9 | 62.9 |  | G28 | L28/T1 | 4.5 | 66.1 |  | G33 | L33/T1 | 1.8 | 64.0 |
| G22 | | L22/T1 | 2.3 | 62.8 |  | G53 | **L46/T2** | 3.9 | 65.0 |  | G27 | L27/T1 | 4.4 | 62.5 |  | G57 | L50/T2 | 1.8 | 66.5 |
| G21 | | L21/T1 | 2.2 | 60.3 |  | G50 | L43/T2 | 3.7 | 66.6 |  | G42 | L6/T2 | 4.3 | 62.1 |  | G17 | L17/T1 | 1.7 | 66.0 |
| G58 | | L51/T2 | 2.2 | 63.4 |  | G8 | L8/T1 | 3.7 | 63.4 |  | G48 | L41/T2 | 4.2 | 61.2 |  | G25 | L25/T1 | 1.7 | 66.0 |
| G59 | | Commercial Check 1 | 2.5 | 61.4 |  | G59 | Check 1 | 3.7 | 66.1 |  | G59 | Commercial Check 1 | 6.1 | 65.8 |  | G59 | Commercial Check k 1 | 1.2 | 64.0 |
| G60 | | Check 2 | 0.7 | 67.1 |  | G60 | Commercial Check 2 | 3.0 | 63.5 |  | G60 | Commercial Check k 2 | 3.3 | 62.2 |  | G60 | Commercial Check 2 | 2.5 | 67.0 |
|  | |  |  |  |  |  |  |  |  |  |  |  |  |  |  |  |  |  |  |
|  | | Grand Mean | 1.8 | 63.0 |  |  |  | 3.4 | 63.8 |  |  |  | 3.5 | 63.3 |  |  |  | 1.4 | 66.6 |
|  | | LSD_0.05_ | 1.6 | 3.0 |  |  |  | 1.3 | 2.5 |  |  |  | 1.9 | 6.5 |  |  |  | 1.7 | 2.0 |
|  | | Heritability | 0.5 | 0.5 |  |  |  | 0.8 | 0.7 |  |  |  | 0.8 | 0.4 |  |  |  | 0.1 | 0.7 |
|  | | Mean of Top 15 hybrid | 2.6 |  |  |  |  | 4.2 |  |  |  |  | 5.0 |  |  |  |  | 2.2 |  |
|  | | Mean of Checks | 1.6 |  |  |  |  | 3.4 |  |  |  |  | 4.7 |  |  |  |  | 1.9 |  |
|  | |  |  |  |  |  |  |  |  |  |  |  |  |  |  |  |  |  |  |
|  | | Advantage of top 15 hybrids over checks | 26% |  |  |  |  | 11% |  |  |  |  | 3% |  |  |  |  | 9% |  |
|  | | Advantage of best hybrid over mean of checks | 36% |  |  |  |  | 20% |  |  |  |  | 14% |  |  |  |  | 30% |  |
|  | | Advantage of best hybrid over the best check | 15% |  |  |  |  | 15% |  |  |  |  | 1% |  |  |  |  | 15% |  |

GY = Grain yield; AD = Days to anthesis

Table 4: Mean performance of 15 high-yielding testcross hybrids and commercial checks across four environments in Uganda, 2016. Entries that are consistent in individual environment and across environments are bolded and underlined

| Genotype No. | Cross | Grain yield | Days to anthesis | Grain texture | Husk cover | Ear aspect |
| --- | --- | --- | --- | --- | --- | --- |
|  |  | (t ha^-1^) | (days) | (1-5) | (%) | (1-5) |
| G39 | **L2/T2** | 3.9 | 63.7 | 3.3 | 15.7 | 2.6 |
| G53 | **L46/T2** | 3.8 | 65.0 | 1.8 | 8.8 | 2.4 |
| G23 | **L23/T1** | 3.6 | 64.8 | 2.1 | 10.2 | 2.3 |
| G52 | **L45/T2** | 3.6 | 63.0 | 2.1 | 12.6 | 2.5 |
| G9 | **L9/T1** | 3.5 | 63.2 | 2.3 | 14.6 | 2.5 |
| G24 | L24/T1 | 3.4 | 66.6 | 2.3 | 13.9 | 2.8 |
| G20 | L20/T1 | 3.4 | 69.2 | 2.5 | 9.0 | 2.2 |
| G28 | L28/T1 | 3.4 | 66.5 | 2.5 | 15.4 | 2.6 |
| G22 | L22/T1 | 3.3 | 64.4 | 2.1 | 15.6 | 2.5 |
| G38 | L38/T2 | 3.3 | 63.6 | 2.8 | 14.8 | 2.4 |
| G43 | L39/T2 | 3.1 | 64.1 | 2.8 | 12.3 | 2.6 |
| G27 | L27/T1 | 3.0 | 63.9 | 3.2 | 19.4 | 2.7 |
| G42 | L6/T2 | 3.0 | 63.0 | 2.7 | 41.0 | 2.8 |
| G29 | L29/T1 | 3.0 | 62.9 | 4.0 | 16.2 | 2.7 |
| G36 | L36/TI | 2.9 | 64.4 | 3.6 | 14.2 | 2.9 |
| G59 | Check 1 | 3.5 | 64.9 | 2.1 | 7.8 | 2.7 |
| G60 | Check 2 | 2.5 | 63.8 | 2.0 | 11.2 | 2.9 |
|  |  |  |  |  |  |  |
|  | Min | 0.3 | 61.3 | 1.6 | 7.8 | 2.5 |
|  | Max | 3.9 | 69.2 | 4.0 | 74.9 | 3.5 |
|  | Grand Mean | 2.3 | 64.2 | 2.7 | 22.7 | 2.8 |
|  | LSD_0.05_ | 0.9 | 1.9 | 0.7 | 24.1 | 0.4 |
|  | Mean of top 15 hybrid | 3.3 |  |  |  |  |
|  | Mean of checks | 3.0 |  |  |  |  |
|  |  |  |  |  |  |  |
|  | Advantage of top 15 hybrids over checks | 5.9% |  |  |  |  |
|  | Advantage of best hybrid over mean of checks | 13.5% |  |  |  |  |
|  | Advantage of best hybrid over the best check | 5.7% |  |  |  |  |

Table 5: Means grain weight loss and number of damaged kernels, weevil mortality and other weevil resistance parameters of the 15 high-yielding testcross hybrids and commercial checks across four environments

| Entry | Cross | Grain weight loss (g) | Number of damaged kernels | Weevil mortality | Number of exit holes | Weight of powder (g) |
| --- | --- | --- | --- | --- | --- | --- |
| G58 | L51/T2 | 2.7^a^ | 21.8^a^ | 2.0^abc^ | 27.0^a^ | 0.1^a^ |
| G16 | L16/T1 | 2.9^a^ | 30.4^abc^ | 1.9^abc^ | 40.6^ab^ | 0.2^a^ |
| G55 | L48/T2 | 3.9^ab^ | 28.4^ab^ | 2.6^abcd^ | 40.4^ab^ | 0.3^a^ |
| G24 | L24/T1 | 4.0^ab^ | 32.8^abcd^ | 4.5^abcde^ | 49.4^abcde^ | 0.3^a^ |
| G49 | L42/T2 | 4.4^ab^ | 41.0^abcdef^ | 2.6^abcd^ | 53.3^abcdefg^ | 0.3^a^ |
| G52 | L45/T2 | 4.8^abc^ | 41.4^abcdef^ | 2.4^abcd^ | 48.0^abcd^ | 0.3^a^ |
| G28 | L28/T1 | 4.9^abcd^ | 59.0^bcdefghij^ | 3.5^abcd^ | 63.1^abcdefghij^ | 0.4^a^ |
| G25 | L25/T1 | 5.2^abcd^ | 37.6^abcdef^ | 5.5^abcde^ | 54.8^abcdefg^ | 0.4^a^ |
| G36 | L36/TI | 5.2^abcd^ | 44.0^abcdefgh^ | 2.8^abcd^ | 60.1^abcdefgh^ | 0.5^a^ |
| G54 | L47/T2 | 5.5^abcd^ | 40.8^abcdef^ | 1.1^ab^ | 66.6^abcdefghijk^ | 0.3^a^ |
| G23 | L23/T1 | 5.5^abcd^ | 48.0^abcdefgh^ | 3.8^abcde^ | 72.1^abcdefghijk^ | 0.4^a^ |
| G56 | L49/T2 | 5.5^abcd^ | 52.0^abcdefghi^ | 10.9^f^ | 73.3^abcdefghijk^ | 0.4^a^ |
| G12 | L12/T1 | 5.5^abcd^ | 50.3^abcdefgh^ | 2.4^abcd^ | 63.5^abcdefghij^ | 0.4^a^ |
| G5 | L5/T1 | 5.8^abcd^ | 52.3^abcdefghi^ | 3.3^abcd^ | 71.0^abcdefghijk^ | 0.5^a^ |
| G40 | L3/T2 | 5.9^abcd^ | 62.0^bcdefghij^ | 2.5^abcd^ | 84.8^bcdefghijklm^ | 0.4^a^ |
| G59 | Check 1 | 5.2^abcd^ | 42.6^abcdef^ | 6.1^bcdef^ | 61.4^abcdefghi^ | 0.3^a^ |
| G60 | Check 2 | 9.4^abcdef^ | 35.4^abcde^ | 1.1^ab^ | 42.0^abc^ | 0.2^a^ |
|  |  |  |  |  |  |  |
|  | Mean | 10.9 | 56.2 | 3.6 | 79.1 | 0.7 |
|  | *P* | *** | *** | NS | *** | NS |
|  | SE | 4.5 | 12.3 |  | 17 |  |
|  | LSD_0.05_ | 12.1 | 34.1 |  | 47.3 |  |
|  | Mean of Top 15 hybrid | 4.8 | 42.8 | 3.4 | 57.9 | 0.3 |
|  | Mean of Checks | 7.3 | 39.0 | 3.6 | 51.7 | 0.3 |

^NS^ not significant, ^*, **, ***^ denotes significant at *P* < 0.05, 0.01, and 0.001 respectively. Means with the same letters in the same column are not significantly different according to Fisher’s Protected LSD.

Table 6: Pearson correlation coefficients among maize weevil resistance parameters for 15 high-yielding testcross hybrids and commercial checks across four environments.

|  | Grain weight loss (g) | Number of damaged kernels | Weevil mortality | Number of exit holes | Weight of powder | Grain Texture |
| --- | --- | --- | --- | --- | --- | --- |
| Grain weight loss (g) |  |  |  |  |  |  |
| Number of damaged kernels | 0.53*** |  |  |  |  |  |
| Weevil mortality | 0.16 | 0.22 |  |  |  |  |
| Number of exit holes | 0.54*** | 0.96*** | 0.22 |  |  |  |
| Weight of powder | 0.12 | 0.20 | -0.03 | 0.19 |  |  |
| Grain texture | 0.20 | 0.16 | 0.26* | 0.11 | 0.12 |  |
| Ear aspect | 0.29* | 0.20 | 0.04 | 0.21 | 0.24 | 0.39** |

*, **, *** denotes significant at P < 0.05, 0.01, an 0.001 respectively

Supplementary Table 1: Heritability variance decomposition and genotype significance for grain yield, agronomic traits and weevil resistance components of 58 testcross hybrids and 2 checks for each environment in Uganda, 2016

| **Statistic** | **Grain yield (GY)** | **Days to anthesis (AD)** | **Husk cover (HC)** | **Grain texture (Tex)** | **Ear aspect (EA)** | **Weevil mortality** | **Kernel damage** | **Number of exit holes** | **Weight of sample** | **Weight of powder (g)** | **Grain weight loss (g)** |
| --- | --- | --- | --- | --- | --- | --- | --- | --- | --- | --- | --- |
| **Bulindi** |  |  |  |  |  |  |  |  |  |  |  |
| Heritability | 0.77 | 0.44 | 0.20 | 0.61 | 0.36 | 0.09 | 0.49 | 0.46 | 0.43 | 0.01 | 0.43 |
| Genotype Variance | 1.52 | 4.10 | 116.80 | 0.38 | 0.04 | 0.86 | 1173.84 | 2210.18 | 27.37 | 0.11 | 27.37 |
| Residual Variance | 0.90 | 10.25 | 914.35 | 0.48 | 0.14 | 17.86 | 2440.63 | 5223.18 | 74.03 | 32.74 | 74.03 |
| Grand Mean | 3.27 | 62.24 | 17.32 | 3.02 | 2.80 | 4.08 | 48.77 | 71.87 | 40.91 | 0.95 | 9.09 |
| LSD | 1.67 | 4.29 | 27.41 | 1.12 | 0.45 | 2.53 | 70.54 | 99.90 | 11.43 | 0.94 | 11.43 |
| CV | 28.07 | 5.06 | 174.58 | 23.08 | 13.43 | 103.67 | 101.30 | 100.56 | 21.03 | 604.77 | 94.70 |
| Genotype significance | 0.00 | 0.02 | 0.38 | 0.00 | 0.09 | 0.73 | 0.02 | 0.03 | 0.04 | 0.98 | 0.04 |
|  |  |  |  |  |  |  |  |  |  |  |  |
| **Ikulwe** |  |  |  |  |  |  |  |  |  |  |  |
| Heritability | 0.00 | 0.59 | 0.09 | 0.65 | 0.00 | 0.56 | 0.50 | 0.50 | 0.57 | 0.63 | 0.57 |
| Genotype Variance | 0.00 | 2.62 | 37.57 | 0.31 | 0.00 | 13.23 | 4465.37 | 8913.13 | 148.09 | 0.09 | 148.09 |
| Residual Variance | 1.07 | 3.62 | 752.91 | 0.34 | 0.37 | 20.61 | 8844.47 | 17753.97 | 223.62 | 0.11 | 223.62 |
| Grand Mean | 1.44 | 66.67 | 30.55 | 2.67 | 3.20 | 4.15 | 119.98 | 170.88 | 33.54 | 0.37 | 16.46 |
| LSD | 0.00 | 2.99 | 16.65 | 0.96 | 0.00 | 6.84 | 134.01 | 189.80 | 22.73 | 0.52 | 22.73 |
| CV | 71.10 | 2.90 | 89.80 | 21.79 | 18.89 | 109.27 | 78.38 | 77.98 | 44.58 | 86.72 | 90.87 |
| Genotype significance | 1.00 | 0.00 | 0.72 | 0.00 | 1.00 | 0.00 | 0.01 | 0.01 | 0.00 | 0.00 | 0.00 |
|  |  |  |  |  |  |  |  |  |  |  |  |
| **Ngetta** |  |  |  |  |  |  |  |  |  |  |  |
| Heritability | 0.44 | 0.18 | 0.32 | 0.35 | 0.50 | 0.00 | 0.00 | 0.00 | 0.00 | 0.29 | 0.00 |
| Genotype Variance | 0.24 | 0.32 | 69.62 | 0.22 | 0.14 | 0.00 | 0.00 | 0.00 | 0.00 | 0.04 | 0.00 |
| Residual Variance | 0.59 | 2.83 | 294.81 | 0.79 | 0.28 | 34.60 | 5024.40 | 11790.18 | 132.52 | 0.20 | 132.52 |
| Grand Mean | 1.75 | 62.97 | 25.59 | 2.77 | 1.58 | 5.53 | 78.10 | 108.27 | 39.76 | 0.56 | 10.24 |
| LSD | 1.04 | 1.46 | 19.81 | 1.07 | 0.75 | 0.00 | 0.00 | 0.00 | 0.00 | 0.48 | 0.00 |
| CV | 43.92 | 2.67 | 67.09 | 32.10 | 33.46 | 106.44 | 90.76 | 100.28 | 28.95 | 78.92 | 112.46 |
| Genotype significance | 0.03 | 0.47 | 0.17 | 0.10 | 0.01 | 1.00 | 1.00 | 1.00 | 1.00 | 0.20 | 1.00 |
|  |  |  |  |  |  |  |  |  |  |  |  |
| **Serere** |  |  |  |  |  |  |  |  |  |  |  |
| Heritability | 0.83 | 0.51 | 0.33 | 0.73 | 0.42 | 0.18 | 0.65 | 0.68 | 0.76 | 0.03 | 0.76 |
| Genotype Variance | 0.84 | 1.26 | 115.33 | 0.23 | 0.04 | 5.06 | 2001.52 | 3854.53 | 63.62 | 0.00 | 63.62 |
| Residual Variance | 0.33 | 2.47 | 469.24 | 0.17 | 0.11 | 44.75 | 2162.61 | 3586.95 | 40.37 | 0.13 | 40.37 |
| Grand Mean | 2.99 | 63.54 | 20.27 | 2.52 | 3.26 | 4.45 | 78.00 | 106.77 | 42.11 | 0.45 | 7.89 |
| LSD | 1.11 | 2.27 | 24.99 | 0.73 | 0.43 | 5.78 | 75.62 | 99.43 | 11.19 | 0.12 | 11.19 |
| CV | 19.34 | 2.46 | 106.85 | 16.43 | 10.13 | 150.18 | 59.62 | 56.09 | 15.09 | 80.66 | 80.51 |
| Genotype significance | 0.00 | 0.01 | 0.13 | 0.00 | 0.04 | 0.44 | 0.00 | 0.00 | 0.00 | 0.91 | 0.00 |
